# Unconventional model organisms bend our view on mitochondrial cristae

**DOI:** 10.1242/jcs.264310

**Published:** 2026-03-06

**Authors:** Silvia Tassan-Lugrezin, Silje A. C. A. Debets, Laura van Niftrik, Taco W. A. Kooij, Irina Bregy

**Affiliations:** ^1^ Department of Medical Microbiology, Radboud University Medical Centre, Nijmegen, 6525 GA, The Netherlands; ^2^ Department of Microbiology, Faculty of Science, Radboud University, Nijmegen, 6525 AJ, The Netherlands

**Keywords:** *Trypanosoma brucei*, *Toxoplasma gondii*, *Plasmodium falciparum*, Apicomplexa, Kinetoplastida, Mitochondrial cristae, ATP synthase, MICOS, Cardiolipin

## Abstract

Cristae, convolutions of the inner mitochondrial membrane, provide an extended surface area for respiratory chain complexes and ATP synthases. Crista structure has been extensively researched in opisthokont model organisms, such as yeast and various animals; however, the vast majority of eukaryotic cristae diversity has been largely unexplored. Here, we provide a comprehensive overview of crista formation and maintenance in Euglenozoa and Alveolata, two highly divergent eukaryotic clades that include parasites of clinical and veterinary importance. Within these clades, cristae have been studied primarily in the kinetoplastid *Trypanosoma brucei* and the apicomplexan *Toxoplasma gondii*. We also discuss the apicomplexan *Plasmodium falciparum*, the deadliest human parasite and etiological agent of malaria, in which *de novo* formation of cristae occurs naturally following an apparently acristate life cycle stage. We compare findings from these divergent and disease-relevant organisms with those from more traditional model organisms, highlighting conserved and unique traits across the eukaryotic kingdom. In this Review, we focus on the roles of three key players in crista curvature – ATP synthase, the mitochondrial contact site and cristae organizing system (MICOS) and cardiolipin, a lipid specific to the inner mitochondrial membrane. By comparing distantly related organisms, we synthesize a broadly applicable model of the general principles of crista formation.

## Teaser

**Summary:** By comparing crista shaping and maintenance across Euglenozoa, Alveolata and Opisthokonta, this Review delineates evolutionarily conserved mechanisms underpinning a general model for crista formation.

## Introduction

**Fig. 1.**
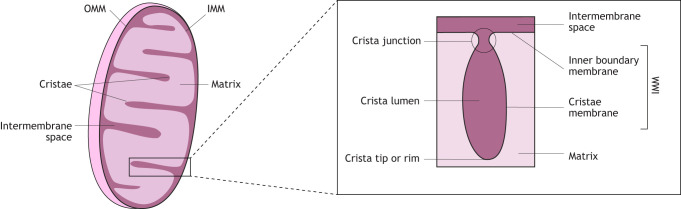
**Schematic introduction to crista structure.** Schematic overview of mitochondrial structure: the OMM serves as a semi-permeable barrier to the cytoplasm. The IMM surrounds the mitochondrial matrix. The IMM is composed of the inner boundary membrane and the cristae membrane. The inner boundary membrane and cristae membrane are connected through crista junctions, areas of high negative curvature of the IMM.

Mitochondria, found in most eukaryotic organisms, are double-membraned organelles with an outer mitochondrial membrane (OMM), which serves as a semi-permeable barrier to the cytoplasm, and an inner mitochondrial membrane (IMM), which surrounds the mitochondrial matrix (Fig. 1). The IMM comprises two domains – the inner boundary membrane domain, which forms contact sites with the OMM to allow protein and lipid transport between the cytosol and the matrix, and the cristae membrane, which forms the majority of the IMM (Panek et al., 2020). The convoluted cristae membrane is specialized for ATP production and highly abundant in the electron transport chain (ETC) and ATP synthase complexes (Gilkerson et al., 2003; Vogel et al., 2006). The inner boundary membrane and cristae membrane are connected through narrow tubular membrane channels known as crista junctions (Frey et al., 2002). Crista junctions are controlled by the mitochondrial contact site and cristae organizing system (MICOS), which influences crista structure by creating negative curvature of the IMM (Rampelt et al., 2017; Stephan et al., 2020; van der Laan et al., 2016). Furthermore, studies in yeast and mammalian cells have shown that formation of rows of ATP synthase dimers creates positive curvature at the rims (or tips) of cristae (Blum et al., 2019; Davies et al., 2012; Paumard et al., 2002; Spikes et al., 2021; Strauss et al., 2008). Additionally, cardiolipin, a phospholipid found in the IMM, has been shown to contribute to crista curvature through multiple mechanisms (Horvath and Daum, 2013; Ikon and Ryan, 2017; Paradies et al., 2019; Venkatraman et al., 2023). Finally, the homologous dynamin-related GTPases optic atrophy 1 (OPA1; mammals) and Mgm1 (yeast) (Delettre et al., 2000), have been shown to contribute to crista curvature. In fact, besides their main function in fusion of the IMM, both OPA1 and Mgm1 have also been implicated in crista stabilization (Amutha et al., 2004; Cipolat et al., 2004; Glytsou et al., 2016; Meeusen et al., 2006; Song et al., 2007). However, these proteins are suggested to be restricted to opisthokonts (Hashimi, 2025; Hashimi et al., 2025).

**Fig. 2.**
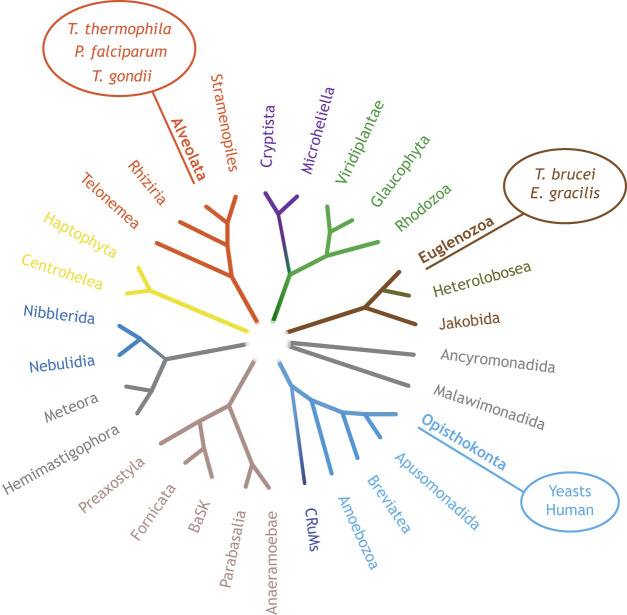
**Schematic overview of the eukaryotic tree of life.** Organism groups discussed in this review article are shown in bold. Yeast and humans belong to the Opisthokonta, *P. falciparum*, *T. gondii* and *T. thermophila* belong to the Alveolata, and *T. brucei* and *E. gracilis* belong to the Euglenozoa. Adapted from Yazaki et al. (2025), where it was published under a CC-BY 4.0 license. BaSK, Barthelona and Skoliomonas; CRuMs, Collodictyonida, Rigifilida and Mantamonadida.

Most review articles describing the formation and curvature of cristae emphasize research in traditional model organisms, such as yeast and mammals, often overlooking the mechanisms underlying crista curvature in other less-studied organisms. This Review aims to address this gap by describing components that influence crista curvature specifically in selected unconventional model organisms (Fig. 2). Unicellular parasites are increasingly being used to answer biological questions, as they offer unique advantages and an evolutionarily distinct point of view compared to traditional model organisms. By comparing the mechanisms of crista curvature between early branching, distantly related species, we generate a model of the basic principle of crista formation that we propose to reflect a general, evolutionarily conserved concept that applies to both traditional as well as unconventional model organisms. Our primary focus is on unicellular parasites that commonly infect humans – *Toxoplasma gondii, Trypanosoma brucei* and *Plasmodium falciparum* – as the mitochondrial functions of these species have been previously studied and they represent some of the most distantly related eukaryotic clades. *T. gondii*, responsible for toxoplasmosis, and *P. falciparum*, the etiological agent of malaria*,* are both Apicomplexa, a large and diverse family of unicellular parasites distantly related to free-living protists, such as dinoflagellates and ciliates (Wolters, 1991). *T. brucei,* the causative agent of human African sleeping sickness, belongs to the Kinetoplastida, a group within the Euglenozoa (Kostygov et al., 2021), which includes both free-living and parasitic species such as *Trypanosoma cruzi*, which causes Chagas disease, and *Leishmania*, responsible for various types of leishmaniasis.

Throughout its complex life cycle, *T. gondii* always possesses a single mitochondrion that contains bulbous cristae (Ferguson and Dubremetz, 2014; Muhleip et al., 2021). These cristae are highly active during the fast-growing life cycle stages (called tachyzoites), in which the parasites rely on oxidative phosphorylation and cause acute toxoplasmosis (Ovciarikova et al., 2017). In contrast, the slow-growing life cycle stages (called bradyzoites, responsible for the formation of persistent tissue cysts) are believed to mainly rely on glycolysis, while mitochondrial energy metabolism is highly reduced (Al-Malki, 2021; Denton et al., 1996).

*T. brucei* also possesses a single mitochondrion that undergoes numerous changes throughout its life cycle (Bily et al., 2021). One major change is a variation in the abundance of cristae between different life cycle stages. In the insect-specific procyclic stages, the mitochondrion is highly active and contains many discoidal cristae to ensure efficient production of ATP (Bringaud et al., 2006; Lamour et al., 2005). The bloodstream form contains significantly fewer cristae, as oxidative phosphorylation is suppressed and the parasite instead depends on substrate-level phosphorylation, mainly by glycolysis, for energy conversion (Kaurov et al., 2018; Zikova, 2022).

*P. falciparum* takes life-cycle-dependent changes in mitochondria to an extreme. Like *T. gondii* and *T. brucei*, the asexual blood stages of *P. falciparum*, responsible for causing pathology, possess a single mitochondrion. Strikingly, electron microscopy (EM) data of single sections of *P. falciparum* and the murine malaria model *Plasmodium berghei* indicate the mitochondrion of asexual blood stages is devoid of cristae as opposed to the cristate mitochondrion found in the sexual blood stages required for transmission to the mosquito vector (Evers et al., 2021, 2025; Sturm et al., 2015; Verhoef et al., 2024). The absence of IMM invaginations was also confirmed in the asexual blood stages of *P. falciparum* using volumetric EM (Evers et al., 2021, 2025; Verhoef et al., 2024). In contrast, the sexual blood stages contain multiple mitochondria in which bulbous cristae form *de novo* (Evers et al., 2021, 2025; Tassan-Lugrezin et al., 2026 preprint).

Within the scope of this Review, we collect and categorize the structural components that have been shown or are expected to be involved in forming and shaping mitochondrial cristae in *T. gondii, T. brucei* and *P. falciparum.* We describe the primary function of each component and compare them to their counterparts in traditional model organisms. By integrating insights from the most divergent eukaryotic clades, we synthesize a model of evolutionarily conserved aspects of crista formation. We propose that this model thus represents a general principle for crista formation across species rather than a detailed map of clade-specific modulators.

## Curvature control and anchoring of the crista junction is mediated by MICOS

MICOS is a multi-subunit protein complex localized in the IMM that is responsible for generating and stabilizing crista junctions as well as creating contact sites in coordination with proteins located in the OMM (Harner et al., 2011; Hoppins et al., 2011; von der Malsburg et al., 2011). In yeast and humans, MICOS is organized into two protein complexes integrated in the cristae membrane – the MIC10 and the MIC60 subcomplexes, both consisting of multiple subunits (Bohnert et al., 2015; Friedman et al., 2015; Guarani et al., 2015). Phylogenetic studies have identified possible orthologs of MIC60 (also known as IMMT in mammals) in *P. falciparum* and *T. gondii* (PfMIC60 and TgMIC60, respectively) (Huynen et al., 2016). Moreover, orthologs of MIC10 (also known as MICOS10 in mammals; TgMIC10) and MIC19 (also known as CHCHD3 in mammals; PfMIC19) have been identified in *T. gondii* and *P. falciparum*, respectively (Munoz-Gomez et al., 2015; Tassan-Lugrezin et al., 2026 preprint). It remains unclear whether the remaining components of MICOS are present in these species. It is possible that identification of additional MICOS components has been obstructed by a high degree of divergence and/or rearrangement of functional domains into poorly annotated genes. Interestingly, we recently reported that PfMIC60 contains a large N-terminal extension of unknown function (Tassan-Lugrezin et al., 2026 preprint). This finding highlights the high divergence of the protein but might also be an indication of functional rearrangement of the components within *P. falciparum* MICOS.

The main component of the MIC10 subcomplex is the protein MIC10, which localizes to the crista junctions and oligomerizes via its intramembrane glycine motifs to induce negative crista curvature (Alkhaja et al., 2012; Barbot et al., 2015; Bohnert et al., 2015). Proteins associated with the MIC10 subcomplex include MIC12, MIC26 and MIC27 for yeast and MIC13 (also known as MICOS13), MIC26 (also known as MIC23 or APOO) and MIC27 (also known as APOOL) for humans (Bohnert et al., 2015; Guarani et al., 2015; Huynen et al., 2016). In both yeast and human cells, the destabilization of the MIC10 subcomplex – in particular through the ablation of MIC10 itself – results in aberrant crista morphology and reduced numbers of crista junctions (Stephan et al., 2020).

**Fig. 3.**
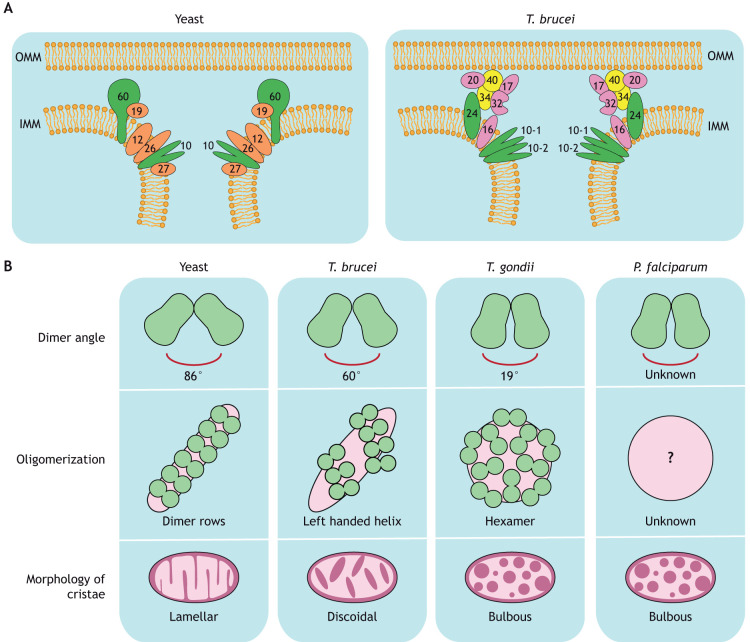
**Schematic overview of crista shaping components in traditional and unconventional model organisms.** (A) The MICOS in yeast (left) and *T. brucei* (right). Conserved proteins directly involved in membrane bending are shown in green, conserved proteins with an indirect or unclear involvement in membrane bending are shown in red, *T. brucei-*specific proteins are shown in yellow if directly involved in membrane bending, or in purple if they have an indirect effect. Numbers refer to the respective MICOS components: MIC60, MIC19, MIC10, MIC12, MIC26 and MIC27 in yeast, and MIC24, MIC34, MIC40, MIC10-1, MIC10-2, MIC20, MIC17, MIC32 and MIC16 in *T. brucei*. (B) Schematic figure of ATP synthase characteristics in yeast, *T. brucei*, *T. gondii* and *P. falciparum*. Dimeric ATP synthase structures have been solved for yeast, *T. brucei* and *T. gondii*, revealing dimer angles of 86°, 60° and 19°, respectively. For *P. falciparum*, the dimer angle is unknown. The different oligomerization states observed in these organisms are associated with different cristae shapes. In yeast, dimer rows produce lamellar cristae; in *T. brucei,* helical arrays of dimers produce discoidal cristae; in *T. gondii*, hexameric oligomerization generates bulbous cristae. The oligomerization state of *P. falciparum* ATP synthase is unknown (indicated by the question mark) but *P. falciparum* has been shown to have bulbous morphology of cristae.

In *T. brucei*, MICOS has been identified but it is highly divergent from both human and yeast MICOS (Fig. 3A). *T. brucei* MIC10 exists as a set of duplicated yet distinct proteins, TbMIC10-1 and TbMIC10-2 (Kaurov et al., 2018). Even though these paralogs show considerable sequence variations compared to the MIC10 of yeast, there is evidence of their involvement in cristae membrane bending. TbMIC10-1 and TbMIC10-2 both localize to the cristae membrane at the crista junctions but deletion of either TbMIC10-1 or TbMIC10-2 individually does not result in a clear change in phenotype of the mitochondria and cristae (Kaurov et al., 2018). However, increased mitochondrial area and elongated cristae are reported when both TbMIC10-1 and TbMIC10-2 are knocked down. In some cases, this also results in loss of the native discoidal cristae shape, and cristae are described as stacked semi-circles or arcs. Interestingly, a 3D reconstruction of serial transmission electron microscopy (TEM) cryosections reveals cristae that lacked crista junctions and instead exhibited a barrel-like shape with finger-like projections in this double knockdown (Kaurov et al., 2018). Overall, we conclude that even though the two MIC10 paralogs are functionally redundant, the presence of at least one of the two is essential for correct organization of cristae and crista junction biogenesis in *T. brucei*.

The MIC60 subcomplex takes its name from its key component MIC60 and it is predicted to be responsible for the negative curvature at the base of the crista membrane (Bock-Bierbaum et al., 2022). In traditional model organisms, MIC60 itself consists of an N-terminal transmembrane domain that anchors to the IMM, a coiled-coil domain and a conserved C-terminal domain, known as the mitofilin domain (Rabl et al., 2009). Both the coiled-coil domain and the mitofilin domain are involved in crista junction formation (Bock-Bierbaum et al., 2022; Tarasenko et al., 2017; Zerbes et al., 2012), but only the latter is essential for forming the narrow tubules of the crista junctions (Korner et al., 2012). The coiled-coil domain has a crucial function in the tetramerization of MIC60, which is an essential process for crista junction stabilization (Bock-Bierbaum et al., 2022). Moreover, the MIC60 C-terminal domain interacts with multiple proteins, such as the OMM protein sorting and assembly machinery (SAM), the translocase of the outer membrane (TOM) and the mitochondrial intermembrane space assembly machine protein MIA40 (also known as CHCHD4 in mammals) (Korner et al., 2012; Ott et al., 2012; von der Malsburg et al., 2011; Xie et al., 2007; Zerbes et al., 2012). The MIC60 complex also includes MIC19 in yeast and paralogs MIC19 and MIC25 (also known as CHCHD6) in humans (Huynen et al., 2016; Munoz-Gomez et al., 2015). Depletion of MIC60 generates similar phenotypes to those observed for MIC10 depletion, with aberrant crista structure, absence of crista junctions and atypical mitochondrial morphology (Stephan et al., 2020; Tarasenko et al., 2017; Zerbes et al., 2012).

In *T. brucei*, the identification of a canonical MIC60 ortholog (TbMIC60) has been ambiguous. In fact, the protein that was first described as TbMIC60 (Kaurov et al., 2018) does not appear to have a mitofilin domain. Instead, recent evidence shows that the ancestral MIC60 is split into two functional parts – the integral membrane protein TbMIC24 (formerly described as TbMIC60) and the two paralogous proteins TbMIC34 and TbMIC40 (Sheikh et al., 2025). TbMIC34 and TbMIC40 are soluble proteins of the intermembrane space, each containing a cryptic mitofilin domain. Moreover, TbMIC34 has also been proven to interact with phospholipids *in vitro* and, both TbMIC34 and TbMIC40 have been shown to be capable of remodeling membranes (Sheikh et al., 2025). RNA interference (RNAi) of TbMIC24 or TbMIC34 results in an increase in crista length, an effect that is more pronounced for TbMIC24 RNAi (Kaurov et al., 2018). Similar to the relationship between MIC10 and MIC60 in canonical systems, ablation of TbMIC24 results in a reduction of the TbMIC10-1 and TbMIC10-2 paralogs. Interestingly, however, the inverse relationship between these components deviates from that in the yeast model. Whereas ablation of MIC10 does not affect MIC60 in yeast and human cells (Bohnert et al., 2015; Stephan et al., 2020), TbMIC10-1 or TbMIC10-2 RNAi both lead to a reduction of TbMIC24, as shown by mass spectrometry (Eichenberger et al., 2019). Data on the dependence of TbMIC10-1 and TbMIC10-2 on TbMIC34 are conflicting, as western blot analysis indicates that knockdown of TbMIC34 decreases TbMIC10-1 and TbMIC10-2 (Kaurov et al., 2018). In contrast, mass spectrometry of TbMIC34 RNAi cells suggests no effect on TbMIC10-1 and TbMIC10-2 (Eichenberger et al., 2019).

Interestingly, it is not only TbMIC34 and TbMIC40 that are localized in the intermembrane space. In fact, in *T. brucei*, MICOS is divided into an integral subcomplex (TbMIC10-1 and -2, TbMIC16 and TbMIC24) and a peripheral subcomplex (TbMIC20, TbMIC32, TbMIC34, TbMIC40 and probably TbMIC17) (Fig. 3A) (Eichenberger et al., 2019; Kaurov et al., 2018). Silencing of TbMIC34, TbMIC40 or TbMIC20 results in a downregulation of all peripheral MICOS components (TbMIC20, TbMIC32, TbMIC34 and TbMIC40) and the inhibition of certain aspects of mitochondrial protein import. In particular, a reduction of intermembrane space proteins and subunits of the respiratory complexes has been reported (Eichenberger et al., 2019; Sheikh et al., 2025).

Overall, *T. brucei* MICOS diverges from the human and yeast MICOS complexes in many aspects (Fig. 3A). Unlike in traditional model organisms, in which MIC10 and MIC60 are described to exist in separate subcomplexes, the two TbMIC10 paralogs and TbMIC24 (replacing the IMM integral part of MIC60) are part of the same integral protein subcomplex responsible for crista curvature. The mitofilin-domain proteins TbMIC34 and TbMIC40 are part of a second, peripheral protein network involved in crista morphology and protein import (Eichenberger et al., 2019; Sheikh et al., 2025). The impact of TbMIC20 is more likely indirectly a result of disruption of mitochondrial protein import (Kaurov et al., 2022), which can ultimately affect crista structure. Because TbMIC20, TbMIC34 and TbMIC40 are part of the same subcomplex, it would be reasonable to assume that TbMIC34 and TbMIC40 affect crista curvature indirectly as well, potentially due to their involvement in protein import. Nevertheless, the presence of cryptic mitofilin domains in TbMIC34 and TbMIC40 strongly indicates that *T. brucei* MICOS has acquired a divergent organization and has split the function of MIC60 into two independent proteins. Therefore, future studies should focus on highlighting the different indirect and direct effects of the peripheral MICOS subcomplex on crista structure, as this could unravel major insights into the divergent evolution of this organism.

Extensive divergence is also evident in *P. falciparum* MICOS, where the extended N-terminus of PfMIC60 deviates from the canonical MIC60 structure and many canonical MICOS components remain elusive (Huynen et al., 2016; Tassan-Lugrezin et al., 2026 preprint). The function of PfMIC60 and PfMIC19 in crista shaping has been recently addressed by our group. Both proteins are only expressed in the cristae-bearing sexual blood stages (Tassan-Lugrezin et al., 2026 preprint). Deletion of both PfMIC19 and PfMIC60 still allows formation of cristae in the sexual stages, but cristae are disorganized with a strong reduction of crista junctions (Tassan-Lugrezin et al., 2026 preprint). Functional dissection of individual domains of PfMIC60 as well as the identification of yet unknown MICOS components in this parasite are essential steps towards fully understanding the overall involvement of MICOS in crista organization.

## ATP synthase oligomerization plays a major role in crista architecture

The F_1_F_O_-ATP synthase, also known as complex V of the ETC, is an IMM protein complex that converts ADP and inorganic phosphate into the energy-rich molecule ATP. ATP production is fueled by the membrane potential generated by the ETC and released by the ATP synthase through translocation of protons out of the crista lumen. Besides this anabolic function, ATP synthase can switch to ATP hydrolysis, thereby inverting directionality of proton pumping and producing ADP and inorganic phosphate (Nakano et al., 2023; Valdebenito et al., 2023). ATP synthase is composed of two multi-protein components, F_O_ and F_1_, the structures of which are highly conserved between yeast and humans. The F_O_ complex consists of the membrane-embedded c ring and subunit a (or 6 in yeast) together with the peripheral stalk, comprises oligomycin sensitivity conferral protein (OSCP) and subunits b, d, e, f, g, h, i, k and A6L (or 8 in yeast) (Lai et al., 2023; Srivastava et al., 2018; Walker and Dickson, 2006). The F_1_ complex is localized in the mitochondrial matrix and is composed of the central stalk (subunits ε, δ and γ) and the catalytic head group (a hexamer consisting of three subunits each of α and β) (Lai et al., 2023; Srivastava et al., 2018; Watt et al., 2010).

Although ATP synthase in general is essential for catalytic activity, the oligomerization state of the enzyme is crucial for correct crista organization. In yeast and mammalian cells, ATP synthase is organized into dimers that are assembled with the two monomers oriented at a species-specific angle (in the range of 70° to 90°), thereby determining how much local positive membrane curvature is generated at the crista rim (Fig. 3B) (Blum et al., 2019; Davies et al., 2012; Paumard et al., 2002; Spikes et al., 2021; Strauss et al., 2008). ATP synthase dimerization has been shown to be regulated by subunits e and g; their absence results in anomalous mitochondrial morphology, including aberrant or absent cristae (Arselin et al., 2004; Paumard et al., 2002). Dimers are further organized into rows that are crucial for generating the overall architecture of the cristae (Anselmi et al., 2018; Kuhlbrandt, 2019; Paumard et al., 2002; Strauss et al., 2008).

In *T. brucei*, ATP synthases are also organized in dimers but, rather than forming long rows, the dimers are arranged into short left-handed helices with a 60° angle between the two F_1_F_O_ complexes, ultimately forming helical arrays along the ridges of discoidal cristae (Fig. 3B) (Gahura et al., 2022; Muhleip et al., 2017). As in traditional model organisms, *T. brucei* subunit g is responsible for dimerization of the ATP synthase. A complete loss of dimerization was observed upon RNAi knockdown of subunit g, but this only partially affected parasite growth and survival, as ATP synthase remained catalytically functional (Gahura et al., 2022). Thin section TEM revealed a reduced number of cristae as well as aberrant crista structure, which correlates well with the phenotype reported in yeast upon depletion of subunit e or g (Gahura et al., 2022; Paumard et al., 2002). Moreover, depletion of a functional analog of subunit e (ATPTb8) results in the formation of aberrant cristae and affects the stability and formation of ATP synthase dimers (Cadena et al., 2021).

*T. gondii* ATP synthase structure and activity has been exclusively studied in tachyzoite life cycle stages. It is also organized in dimers that are composed of one F_O_ subcomplex that binds two F_1_F_O_ complexes at an angle of 19°, resulting in milder membrane curvature (Fig. 3B) (Muhleip et al., 2021). Two apicomplexan-specific coiled-coil-helix-coiled-coil-helix (CHCH) proteins, ATPTG8 and ATPTG9, are involved in the dimerization process (Usey and Huet, 2023). CHCH domain proteins have been previously described as serving as a scaffold that structurally supports specific mitochondrial complexes, such as MICOS, and participating in the assembly of respiratory complexes (Cavallaro, 2010; Longen et al., 2009; Modjtahedi et al., 2016). Interestingly, CHCH proteins have not previously been identified as part of the ATP synthase of other organisms (Cavallaro, 2010; Longen et al., 2009). Conditional knockdowns of ATPTG8 and ATPTG9 in *T. gondii* destabilizes ATP synthase through disruption of its dimeric state and allows ions to leak across the IMM (Usey and Huet, 2023). Furthermore, TEM has revealed significantly lower density of cristae in knockdown conditions (Usey and Huet, 2023). In *T. gondii*, ATP synthase dimers are subsequently organized into hexamers that further organize into pentagonal pyramids, which induce additional crista curvature. The role of hexamer formation on crista structure has been investigated with a particular focus on the protein ATPTG11, an apicomplexan-specific F_O_ subunit. ATPTG11 functions as a linker that holds together the dimers involved in hexamer formation. Cellular cryo-electron tomography (cryo-ET) of tachyzoites lacking ATPTG11 shows that the dimers are no longer arranged in pentagonal pyramids but instead are loosely structured in a row-like arrangement (Muhleip et al., 2021). Moreover, mitochondrial cristae in the ATPTG11 knockout are described as having an elongated tubular shape, indicating that ATP synthase hexamers and pentagonal pyramids are essential to maintain the bulbous crista morphology of *T. gondii* (Muhleip et al., 2021).

Likewise, ATP synthases in *P. falciparum* are assembled in dimeric supercomplexes, but the exact organization of the dimers and the players involved in the assembly process are unknown (Fig. 3B) (Nina et al., 2011). Interestingly, complexome profiling identified a homolog of ATPTG9 in *P. falciparum* ATP synthase, although the involvement of this protein in dimerization has not yet been investigated (Evers et al., 2021). A possible higher-order oligomerization state of ATP synthase in *P. falciparum* has also been suggested, possibly matching the hexametric state identified in *T. gondii* (Evers et al., 2021). However, more evidence is needed to confirm the exact type of ATP synthase assembly in malaria parasites.

Interestingly, some studies imply possible interplay between ATP synthase and MICOS; more specifically, mature ATP synthase dimers strongly interact via subunit e and g with MIC10 in yeast, implying possible dual functioning of this MICOS component at both ends of cristae (Rampelt et al., 2022). Remarkably, in *T. brucei,* the functional analog of subunit e (ATPTb8) has been demonstrated as interacting with TbMIC10-1 (Cadena et al., 2021). In contrast, depletion of both TbMIC10-1 and TbMIC10-2 did not affect the expression levels of ATP synthase subunits (Cadena et al., 2021).

Although there are differences in ATP synthase dimerization and higher-order oligomerization states between the divergent eukaryotic clades, crista shaping through ATP synthase supercomplexation appears to be conserved across three of the most diverse eukaryotic clades (i.e. Opisthokonta, Alveolata and Euglenozoa). Furthermore, the interaction of ATP synthase with MIC10, both in traditional and unconventional model organisms, demonstrates a remarkable interplay between the oligomers of ATP synthase and MICOS. Better understanding of this interplay could help unravel new insights and aspects of crista shaping and organization. More research regarding ATP synthase oligomerization and its impact on crista morphology could strengthen this hypothesis and confirm that ATP synthase has an intrinsic and direct role in crista shaping.

## The role of ETC supercomplexes in crista shaping

In addition to their crucial role in the ETC and oxidative phosphorylation, complexes I, III and IV (and rarely complex II) of the ETC have been shown to assemble into supercomplexes, such as supercomplexes I–III2, III2–IV and I–III2–IV in mammals, yeast and plants (Davies et al., 2018; Gu et al., 2016; Letts et al., 2019, 2016; Maldonado et al., 2023; Vercellino and Sazanov, 2021). *In situ* structural analysis of mammalian ETC supercomplexes shows that although some ETC assemblies are associated with minor local membrane curvature, the overall membrane geometry remains largely planar, matching the flat region along the cristae membrane (Zheng et al., 2024).

The potential relevance of this geometry becomes evident upon comparison of conventional model organisms to organisms with diverging cristae shapes. Therefore, the role of ETC supercomplexes in crista morphogenesis and membrane bending activity has been mainly studied in unconventional model organisms. For example, molecular dynamic simulations in *Euglena gracili*s, a free-living flagellate of the Euglenozoa, have shown that supercomplexes I–III2–IV and III2–IV2 have a distinctive organization that adapts to the negative curvature along the *E. gracilis* cristae membrane (He et al., 2024).

Furthermore, several cryo-electron microscopy and tomography studies along curved regions of cristae in *Tetrahymena thermophila*, a free-living ciliate belonging to the alveolates (the clade that also includes the Apicomplexa), have reported the discovery of bent assemblies of ETC supercomplexes (Han et al., 2023; Muhleip et al., 2023; Wolters, 1991; Zhou et al., 2022). Moreover, the *T. thermophila* I–II–III2–IV2 supercomplex has been shown to displace the membrane from its original plane in a molecular dynamics simulation, highlighting the ability of the supercomplex to shape membranes (Muhleip et al., 2023).

Supercomplexes similar to the one reported in *T. thermophila* cannot form in Apicomplexa, in which the canonical complex I has been replaced by a monomeric type II NADH dehydrogenase (Danne et al., 2013; Krungkrai et al., 2002; Lin et al., 2011). Still, complexome profiling suggests the presence of supercomplexes with different ratios of complex III and/or IV in *P. falciparum* and *T. gondii* (Evers et al., 2021; MacLean et al., 2025). Supercomplex III2–IV from *T. gondii* is kinked and localizes to the curved lateral region of mitochondrial cristae (MacLean et al., 2025). However, in *T. gondii,* ablation of apicomplexan-specific COX10, a subunit of complex IV involved in formation of the III2–IV supercomplex, results in the loss of ETC supercomplexes without affecting morphology or density of cristae (MacLean et al., 2025).

A role of individual ETC complexes (rather than supercomplexes) in crista morphology has been suggested in *P. falciparum* based on the depletion of the universally conserved complex III subunit Rieske (PfRieske). Parasites deficient in PfRieske show defects during both asexual and sexual blood-stage development (Sheokand et al., 2024). Considering the apparent absence of cristae in the asexual blood stage, this observation does not immediately indicate involvement of PfRieske in the formation of cristae. Nevertheless, TEM has revealed that the PfRieske knockdown parasites show reduced abundance of cristae in sexual stages (Sheokand et al., 2024).

Based on these contrasting data in different unconventional model organisms, we conclude that, although ETC supercomplexation is involved in stabilizing or refining crista shape, it remains elusive whether it is a major contributor to crista curvature across clades.

## Other potential modulators of crista morphology

Although ATP synthase and MICOS are considered the primary components involved in shaping cristae, additional proteins have been proposed to modulate and refine the formation and architecture of cristae in both traditional and unconventional model organisms.

ATPase inhibitory factor 1 (IF_1_; also known as ATP5IF1 in mammals) is an inhibitor of the ATP synthase found throughout eukaryotes (Hashimoto et al., 1981; Panicucci et al., 2017; Pullman and Monroy, 1963; Usey et al., 2024 preprint). To perform its inhibitory activity, IF_1_ binds the catalytic interface of the ATP synthase in the F_1_ domain (Gledhill et al., 2007). Initially, in animal and yeast cells, IF_1_ was reported to bind ATP synthase to prevent ATP hydrolysis in non-favorable conditions, such as low pH or hypoxia (Cabezon et al., 2002; Campanella et al., 2008; Chimeo et al., 2015; Garcia-Bermudez and Cuezva, 2016). More recently, in mouse tissue cells, IF_1_ has been shown to colocalize with ATP synthase at the tip of cristae and to play a role as a modulator of ATP synthase activity in respiring mitochondria (Romero-Carraminana et al., 2023). In the same study, IF_1_ was reported to be involved in ATP synthase oligomerization and its depletion resulted in crista defects. Furthermore, IF_1_ overexpression in cancer cells leads to an increase in ATP synthase oligomeric assemblies accompanied by a higher density of mitochondrial cristae (Campanella et al., 2008; Faccenda et al., 2017; Garcia-Bermudez and Cuezva, 2016; Gore et al., 2022). However, the data on the role of IF_1_ is still controversial and several studies have failed to agree on the proposed IF_1_-mediated mechanism for ATP synthase oligomerization and remodeling of cristae (Barbato et al., 2015; Fujikawa et al., 2012; Kahancova et al., 2018, 2020).

In *T. gondii*, the homolog of IF_1_ (TgIF_1_) has been shown to bind to ATP synthase dimers (Muhleip et al., 2021). Interestingly, overexpression of TgIF_1_ has also been shown to promote higher-order ATP synthase oligomerization (Usey et al., 2025). Furthermore, TgIF_1_ overexpression leads to improved recovery from oxidative stress, suggesting that this protein might be important for structural or metabolic adaptations to the changing cellular environment throughout the life cycle of the parasite (Usey et al., 2025). As previously described for mammalian IF_1_ (Romero-Carraminana et al., 2023), the ablation of TgIF_1_ also results in a decrease in the density of cristae but no growth defect under normal culture conditions (Usey et al., 2025).

An IF_1_ homolog (TbIF_1_) has also been identified in *T. brucei*. Currently, no data on the effects of TbIF_1_ depletion on crista morphology or on its effect on ATP synthase oligomerization is available. However, it has been reported that TbIF_1_ is differentially expressed between life cycle stages, such that it only appears in the procyclic stage, in which cristae are highly abundant, in order to mediate oxidative phosphorylation (Gahura et al., 2018; Panicucci et al., 2017). The exclusive expression of TbIF_1_ in cristae-rich life cycle stages might support the possibility of it having relevance in the formation, maintenance or function of cristae that might be analogous to its homologs in other species.

However, owing to the natural downregulation of IF_1_ in some cristae-bearing cell types in mammals (Esparza-Molto et al., 2019; Romero-Carraminana et al., 2023) and the controversial data on its role in ATP synthase oligomerization, we hypothesize that IF_1_ is important but not essential for the formation of cristae and to be mainly involved in regulating ATP synthase stability. Interestingly, IF_1_-induced oligomerization of ATP synthase leads to inhibition of its enzymatic activity, suggesting that IF_1_ might regulate both ATP production and crista structure by creating a pool of dormant ATP synthase complexes at the crista rims (Gu et al., 2019; Romero-Carraminana et al., 2023).

The adenine nucleotide translocase (ANT) is a group of closely related isoforms of mitochondrial transport proteins belonging to the mitochondrial carrier family, also known as the solute carrier family 25 (SLC25), located in the IMM and conserved across eukaryotes (Klingenberg, 2008; Kunji et al., 2020). ANT is responsible for transporting ADP and ATP across the IMM (Klingenberg, 2008; Kunji et al., 2016). Although the role of ANT in respiration has been studied in yeast, its effect on crista curvature was not the focus of the study (Pereira et al., 2007). Interestingly, depletion of the *T. gondii* homolog TgANT decreases the number and density of cristae (Qian et al., 2023; Wu et al., 2022). In *T. brucei*, depletion of TbANT results in a decrease in cytosolic ATP combined with a reduced consumption of oxygen in the mitochondria; however, TbANT involvement in crista curvature has not been addressed (Gnipova et al., 2015; Pena-Diaz et al., 2012). Further research is therefore needed to explore whether ANT is directly involved in crista morphology.

Membrane-associated progesterone receptor (MAPR) proteins in mammalian and yeast cells localize in the nucleus, cytoplasm, endoplasmic reticulum and mitochondria, and are primarily known for their involvement in heme or steroid binding and autophagy (Kimura et al., 2012; McGuire and Espenshade, 2023). Although no reports in mammals and yeast have investigated mitochondrial phenotypes associated with MAPR, in *T. gondii* TgMAPR has been implicated in crista shaping. Interestingly, TgMAPR-deficient parasites demonstrated reduced fitness and reduced levels of steroids, and their cristae exhibited an enlarged and disorganized phenotype, indicating possible involvement of TgMAPR in cristae membrane maintenance (Asady et al., 2023). However, further studies of direct orthologs of TgMAPR as well as a detailed study of its function are needed to clarify whether the observed effects are of direct nature or an indirect result of defects in the biogenesis of heme-containing proteins of the ETC and the resulting disruption of respiratory chain complexes and oxidative phosphorylation. We hypothesize that these observed defects in mitochondrial biogenesis and crista structure might be attributed to general effects of stress due to potential disruption of steroid-related processes (Asady et al., 2023).

## Cardiolipin modulates mitochondrial membrane curvature from within

Cardiolipin is a mitochondrial-specific phospholipid that makes up 15–20% of the lipid content of the IMM in yeast, plants and mammals, compared to only 5–10% of the OMM (Horvath and Daum, 2013; Paradies et al., 2019; Schiaffarino et al., 2022). This unique negatively-charged lipid consists of two phosphatidic acids connected by a short glycerol bridge, giving it a unique cone-like shape, with the head smaller than the four acyl chain tails (Lewis and McElhaney, 2009; Schlame, 2008).

Modeling studies have predicted that cardiolipin has a direct effect on crista curvature, a finding which has been demonstrated in yeast. In yeast, cardiolipin was found to act as a buffer against loss of curvature in the IMM (Venkatraman et al., 2023). As a result of its cone-like shape, cardiolipin has an intrinsic negative curvature; thus, it is predicted to localize and cluster in negatively curved areas of the membrane (Beltran-Heredia et al., 2019; Elmer-Dixon et al., 2019; Ikon and Ryan, 2017). Further studies in computational models of mammalian and *Drosophila melanogaster* membranes predict that cardiolipin is not limited to negative curvature regions but can also be found in areas of positive curvature (Konar et al., 2023). In this model, cardiolipin-mediated curvature is determined by the distribution of cations inside the organelle. Owing to its negative charge, cardiolipin can interact with cations, which partially neutralize its charge and modulate membrane packing (Lewis and McElhaney, 2009). In negatively curved regions, cardiolipin promotes negative membrane curvature due to the high concentration of cations, which are electrostatically attracted to the high density of the negative charges of cardiolipin and allow lipids to pack more closely. In positively curved regions, cations are more dispersed, diminishing their impact on cardiolipin geometry and resulting in more fluid and less compact membrane areas (Konar et al., 2023). The involvement of ions in cardiolipin membrane curvature is also supported by experimental results in liposomes – locally lowering pH and therefore increasing cation levels induces crista-like invaginations in liposomes containing cardiolipin but not in liposomes lacking cardiolipin (Khalifat et al., 2011, 2008). Moreover, studies in yeast and *D. melanogaster* as well as molecular dynamics simulations indicate that cardiolipin stabilizes supercomplexes of the ETC, namely complex III, IV and ATP synthase (Acehan et al., 2011; Bazan et al., 2013; Makowski et al., 2025; Pfeiffer et al., 2003; Zhang et al., 2005). Moreover, cardiolipin has been shown to stabilize MIC10 oligomers in yeast (Rampelt et al., 2018).

Based on its presence at specific positions along the interface of ATP synthase dimers, cardiolipin has also been suggested to have an essential function in stabilizing dimer assembly of ATP synthase in *T. gondii* and *T. brucei* (Gahura et al., 2022; Muhleip et al., 2021). The impact of cardiolipin depletion on ATP synthase dimerization and oligomerization in *T. gondii* has not yet been investigated, highlighting the need for further studies in this area. In contrast, depletion of cardiolipin synthase has been shown to strongly affect ATP synthase dimerization in *T. brucei* as a result of downregulation of F_1_F_O_ subcomplex genes (Serricchio et al., 2021). Cardiolipin synthesis is essential for *T. brucei* and its depletion also affects other respiratory chain complexes, such as complex III and IV (Serricchio and Butikofer, 2012; Serricchio et al., 2021). Moreover, TEM of *T. brucei* parasites upon ablation of cardiolipin synthase has revealed an aberrant IMM and loss of cristae (Schadeli et al., 2019).

To conclude, cardiolipin has multiple roles in crista curvature. Cardiolipin has an indirect and conserved effect on crista curvature due to its role in stabilizing ATP synthase dimers and oligomers as well as a direct, pH-dependent impact on membrane curvature. The pH dependency of cardiolipin-mediated membrane invagination is in line with the proton gradient that is actively generated and used during mitochondrial ATP production. Therefore, we hypothesize that metabolic activity of the ETC promotes and stabilizes cardiolipin-induced curvature. Given the conserved structure of cardiolipin, it is reasonable to assume that its direct role also extends to Alveolata and Euglenozoa.

## Synthesis of a general mechanism to explain crista shaping in all organisms

Proper folding and architectural maintenance of cristae is a complex process, and its success relies on the balance of multiple components. In this Review, we have summarized the main factors involved in crista shaping in the unconventional model organisms *T. gondii, P. falciparum* and *T. brucei* and integrated these findings into the context of the extensive research on cristae that has been performed in traditional model organisms.

Careful analysis of the differences and similarities in the proteins and protein complexes involved in forming cristae, as well as the integration of knowledge from traditional model organisms with studies performed on these divergent species, allows for a broader and more complete overview of the general principles of crista formation. The key components essential for crista shaping must be conserved throughout different clades and be observed to have clear crista-shaping activity in all organisms in which they have been studied. Homologs of several key crista-shaping proteins from yeast and humans have been identified in *T. gondii, P. falciparum* and/or *T. brucei*. Still, many other components known to contribute to cristae in opisthokonts are either absent or highly divergent across the distant eukaryotic clades these species represent. Although research in organisms outside of the Opisthokonta is still limited, the presence of some core MICOS components, as well as the shared involvement of ATP synthase and ETC supercomplexes in crista shaping, clearly point towards an overarching principle of crista formation. Evidence suggests that not only is a membrane rich in cardiolipin a favorable canvas for cristae but that cardiolipin actively contributes to the formation of cristae in both traditional and unconventional model organisms. In contrast to the apparently shared involvement of MICOS, ATP synthase, ETC supercomplexes and cardiolipin, we hypothesize that the supporting modulators IF_1_, MARP proteins and ANT are not essential for the general conserved process of crista shaping but rather involved in generating species-specific crista morphology.

**Fig. 4.**
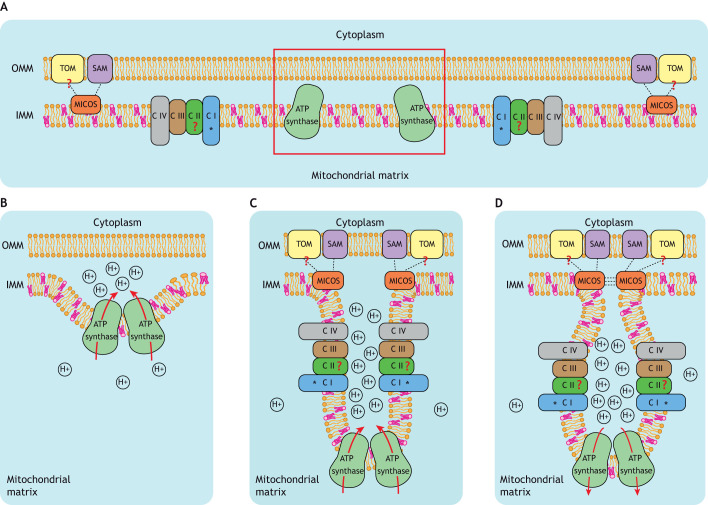
**Schematic representation of the proposed model for crista formation through membrane invagination and outgrowth.** (A) The IMM is enriched in cardiolipin (shown in pink), which primes the membrane for bending due to its unique cone-like structure. In this model, all the protein complexes necessary for crista formation are present prior to the start of membrane bending. Complex I of the ETC (CI) is marked with an asterisk (*) to denote its absence in Apicomplexa, which instead present a distinct NADH dehydrogenase. TOM and complex II (CII) are marked with a red question mark to indicate the uncertain interaction of TOM with MICOS and the unproven presence of CII in ETC supercomplexes (except in *T. thermophila*). The red box indicates the future site of crista formation. (B) Dimerization of ATP synthase complexes generates the initial curvature in the IMM. Under appropriate conditions, ATP synthase performs ATP hydrolysis, thus locally increasing proton concentration in the intermembrane space. (C) The increased concentration of protons at the IMM in proximity to ATP synthase promotes stronger cardiolipin-mediated membrane bending. This generates an unorganized crista in which ATP synthase is positioned at the point of the strongest positive curvature, while CI (*), CII, complex III (CIII) and complex IV (CIV) are passively organized along the rim of the growing crista. (D) Finally, the negative curvature at the crista neck helps position MICOS at this bent region. The intrinsic ability of MICOS to curve membranes allows for further narrowing and shaping of the crista junction, which in turn enables MIC60 tetramerization and finally stabilization of the crista. Interactions between MICOS and the OMM complexes SAM and potentially TOM ensure anchoring of the crista to the OMM.

By integrating all the information collected in this Review, we propose a general mechanism to explain crista formation through membrane invagination and outgrowth (Fig. 4). This model encompasses four main concepts. First, cardiolipin is essential to prime the IMM for crista bending (Panek et al., 2020). Considering that cardiolipin-containing membranes spontaneously invaginate upon local acidification (Khalifat et al., 2011, 2008), cardiolipin offers a potential mechanism for crista formation that is independent of membrane-bending proteins. Still, such proteins are important to provide local acidification, support deep and stable invagination and further refine crista shape. Therefore, the second key contributor is ATP synthase. The natural tendency of ATP synthase to dimerize generates a small initial bending of the membrane. Under appropriate conditions (such as high glucose levels, which support ATP production through glycolysis), ATP synthase can perform ATP hydrolysis, thereby locally acidifying the intermembrane space. This acidification is crucial because it promotes cardiolipin-dependent invagination at the exact location of ATP synthase dimers (Fig. 4B). As the crista grows in an acidification-dependent manner, ATP synthase molecules encounter suitable membrane curvature to allow for higher-order oligomerization. An unorganized crista forms with ATP synthases driving proton accumulation at the crista rim, and an increasing amount of membrane is dragged into the crista, including ETC complexes that follow passively (Fig. 4C). When enriched in the crista membrane, ETC complexes encounter suitable curvature to form supercomplexes, which further facilitates their accumulation. Additionally, the ETC complexes intensify the acidification by participating in proton pumping, thus further inflating the crista. Fourth and finally, passive diffusion pulls previously assembled MICOS towards the future neck of the crista, where MICOS itself contributes to further narrowing and shaping of the crista junction (Fig. 4D). Interaction between MICOS complexes via tetramerization of MIC60 stabilizes the crista junction to ensure contact between the IMM and the OMM (via SAM and TOM complexes) and allow for further stabilization and organization of the crista. Once fully assembled and locked by supercomplexation of ATP synthase, ETC complexes and MICOS, ATP synthesis can safely be initiated without resulting in collapse of the nascent crista.

Future research is essential to clarify the involvement and conservation of the various crista-shaping components. In particular, ATP synthase and its oligomerization states and factors should be investigated in depth in species such as *T. brucei* and *P. falciparum* to identify the key components necessary for positive membrane bending and to confirm that different oligomerization states greatly influence crista morphology. In addition, the data should also be compared and integrated with findings from other unconventional model organisms, such as the green algae *Chlamydomonas reinhardtii* and *Polytomella*, in which mitochondrial cristae and their components have also been partially studied (Dietrich et al., 2024; Lapaille et al., 2010; Waltz et al., 2025). Finally, it would be interesting to test our model in the recently published integrative computational ‘living’ model of human cristae (Brown et al., 2025). This computational model allows for various proteins or complexes to be virtually introduced into a model of a bent membrane, which is then permitted to rearrange itself in accordance with the computationally or experimentally validated properties of the components (Brown et al., 2025). This approach would allow for verification of the roles of the evolutionarily conserved proteins, which we propose to be essential for crista formation.

Malaria parasites, being the only organism known to live through a life cycle stage with apparent absence of cristae followed by *de novo* formation of cristae during sexual maturation (Evers et al., 2021, 2025; Verhoef et al., 2024), have proven to be an extremely insightful tool for understanding cause–consequence relationships during crista growth and maturation (Tassan-Lugrezin et al., 2026 preprint). The model we propose for the formation of cristae based on ATP hydrolysis performed by ATP synthase also supports the timeline for *de novo* formation of cristae in *P. falciparum*. This process occurs while the parasite resides in the human body, where glucose is high enough to support the generation of ATP through glycolysis, ensuring the appropriate conditions necessary for growth and maturation of cristae. Moreover, our model supports the dispensability of MICOS during the initial formation of cristae and, at the same time, its essentiality in correct crista stabilization and shaping (Tassan-Lugrezin et al., 2026 preprint).

## Conclusions

Although mitochondrial cristae are a shared feature of most eukaryotic cells with mitochondria, the limited number of studies on organisms outside the Opisthokonta represents a limitation for the comprehensive understanding of crista morphology (Hashimi, 2019). Using protist parasites to study conserved biological principles has become a common strategy that has already led to major discoveries. In fact, some of the biggest discoveries in molecular biology, such as RNA editing, antigenic variation, glycosylphosphatidylinositol anchoring and trans-splicing, have been discovered and heavily studied in euglenozoan and alveolate organisms, thus establishing them as (unconventional) model organisms (Benne, 1989; Borst and Cross, 1982; de Almeida et al., 1988; Ferguson and Cross, 1984; Lukes et al., 2023; Scherf et al., 2008; Sutton and Boothroyd, 1986). Undoubtedly, much remains to be uncovered in their complex biology that could shed light on evolutionarily conserved processes.

Therefore, we are convinced that identifying additional candidates that directly influence crista curvature in *T. brucei*, *T. gondii* and *P. falciparum* will be crucial to improving knowledge of the molecular basis of crista shaping and architecture. These unconventional model organisms will enable a better understanding of the key components of crista organization and maintenance. Unraveling the phenotypic effects of species-specific differences and determining which aspects retain high evolutionary conservation will deepen insights into crista formation beyond individual species and clades, towards a universal concept true for all cristae-bearing organisms.
